# The dynamic cilium in human diseases

**DOI:** 10.1186/1755-8417-2-3

**Published:** 2009-05-13

**Authors:** Anna D'Angelo, Brunella Franco

**Affiliations:** 1 Telethon Institute of Genetics and Medicine (TIGEM), Naples, Italy; 2 Medical Genetics, Department of Pediatrics, Federico II University, Naples, Italy

## Abstract

Cilia are specialized organelles protruding from the cell surface of almost all mammalian cells. They consist of a basal body, composed of two centrioles, and a protruding body, named the axoneme. Although the basic structure of all cilia is the same, numerous differences emerge in different cell types, suggesting diverse functions. In recent years many studies have elucidated the function of 9+0 primary cilia. The primary cilium acts as an antenna for the cell, and several important pathways such as Hedgehog, Wnt and planar cell polarity (PCP) are transduced through it. Many studies on animal models have revealed that during embryogenesis the primary cilium has an essential role in defining the correct patterning of the body. Cilia are composed of hundreds of proteins and the impairment or dysfunction of one protein alone can cause complete loss of cilia or the formation of abnormal cilia. Mutations in ciliary proteins cause ciliopathies which can affect many organs at different levels of severity and are characterized by a wide spectrum of phenotypes. Ciliary proteins can be mutated in more than one ciliopathy, suggesting an interaction between proteins. To date, little is known about the role of primary cilia in adult life and it is tempting to speculate about their role in the maintenance of adult organs. The state of the art in primary cilia studies reveals a very intricate role. Analysis of cilia-related pathways and of the different clinical phenotypes of ciliopathies helps to shed light on the function of these sophisticated organelles. The aim of this review is to evaluate the recent advances in cilia function and the molecular mechanisms at the basis of their activity.

## The world of cilia

Cilia are dynamic organelles projecting from the cell surface. They consist of a basal body located under the cell surface and of a projecting structure called the axoneme. The basal body is composed of a pair of centrioles embedded in the pericentriolar material (PCM). The ciliary axoneme contains nine microtubule doublets surrounded by a membrane contiguous with the plasma membrane. Cilia are classified on the basis of structure and function. The basic structure of the different types of cilia is similar although their function may be tissue-specific and may change during development, tissue morphogenesis and homeostasis.

**Figure 1:**
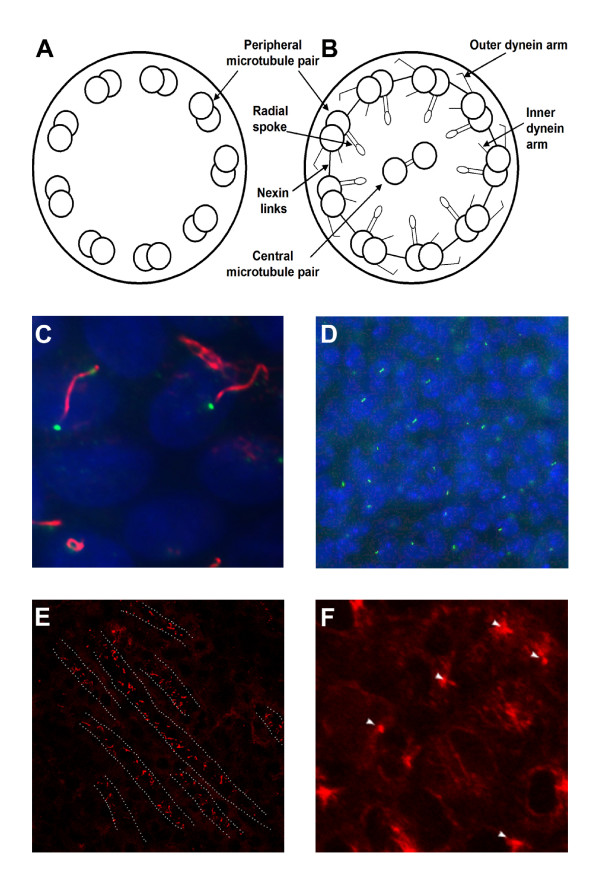
**Cilia with different structures and primary cilia in diverse cell types and tissues**. (A) Cross section of the 9+0 axonemal structure of the non-motile primary cilium. (B) Cross section of the 9+2 axonemal structure of the motile cilium with the motor molecules. (C) Primary cilia on Madin-Darby canine kidney (MDCK) cells. Cilia were stained with anti-α tubulin acetylated (red), basal bodies with anti-γ tubulin (green) and nuclei with 4',6-diamidino-2-phenylindole (DAPI). (D) Primary cilia in the ganglionic eminence of a brain at E12.5. Cilia were revealed by anti-adenylyl cyclase III (green), a marker specific for neuronal cilia, and nuclei were stained with DAPI. (E) Primary cilia in the renal tubules of a mouse at P7. Cilia were stained with anti-α tubulin acetylated (red) and dashed lines indicate the shape of the tubules. (F) Primary cilia in the limb bud in a mouse at P0. Cilia (indicated by arrows) were revealed with anti-α tubulin acetylated (red).

The traditional classification of cilia into two main classes, motile 9+2 and non-motile 9+0, is insufficient to reflect the complexity of all cilia types. The most recent studies indicate that cilia can be divided into at least four main cilia types: motile 9+2, motile 9+0, non-motile 9+2 and non-motile 9+0. In the 9+2 configuration the axoneme contains a central microtubule pair surrounded by the nine microtubule doublets, which is missing in the 9+0 configuration. Moreover, motile cilia contain inner and outer dynein arms, radial spokes and nexin links (reviewed in [1]). Inner and outer dynein arms on the doublets mediate axoneme motility. The radial spokes play an essential role in the control of dynein arm activity by relaying signals from the central pair of microtubules to the arms. Nexin links are the connecting links between microtubules in cilia and flagella. Radial cuts of 9+0 and 9+2 axonemal structures of non-motile and motile cilia, respectively, are illustrated in Figure 1A and 1B. Respiratory and ependymal cilia are motile 9+2 with a back-and-forth motion. In the murine embryonic node, primary cilia are an admixture of 9+2 and 9+0 cilia [2]. The specific feature of the unique 9+0 motile cilium is the rotational movement generating an anti-clockwise flow of extra-embryonic fluid in the nodal area [3]. Thus, the central microtubule pair seems to be required for back-and-forth movement while its absence produces a rotational movement. Non-motile 9+2 cilia are present on specialized olfactory neurons [4]. Renal monocilia, photoreceptor-connecting cilia and cilia of pancreatic islets are non-motile 9+0 cilia [5-7]. Finally, among the different cilia types, 9+4 cilia have been identified on the notochordal plate of the rabbit embryo [8].

The number of cilia can vary among cell types. Epithelial cells may possess several hundred 9+2 motile cilia while 9+0 cilia are usually solitary. Non-motile cilia are generally considered chemical or mechanical sensors and are called 'primary cilia', but recent advances have suggested that all cilia might have sensory functions. The primary cilium only assembles when cells are not in mitosis, and it is considered an organelle of cells in a quiescent or differentiated state. In fact, cell cycle re-entry is preceded by cilium reabsorption [9]. The primary cilium is considered a highly dynamic organelle both because it is assembled only during a certain phase of cell life and because an active molecular transport occurs within its axoneme.

During ciliogenesis, cilia elongate from the basal body through the addition of new axonemal subunits organized in macromolecular particles to the distal tip. Intraflagellar transport (IFT) is responsible for this transfer, which can be bidirectional [10]. Anterograde transport is driven by heterotrimeric kinesin 2, which is composed of two motor subunits (Kif3a and Kif3b) and a non-motor subunit [11]. Retrograde transport back to the cell body is accomplished by cytoplasmic dynein 1B [12,13]. Ciliogenesis is also coordinated by PCM, which functions as a nucleation site for microtubules. RNA interference (RNAi) knockdown of pericentrin, a protein important for PCM organization, inhibits ciliogenesis and reduces the presence of IFT components near the centrioles [14].

Primary cilia are present on a wide variety of cell types such as the bile duct, the kidney tubule, the endocrine pancreas, the thyroid, smooth muscle cells, neurons, fibroblasts, and chondrocytes. Some examples of cells or tissues presenting primary cilia are illustrated in Figure 1C–F. For a complete list of cells and tissues containing cilia, refer to [15].

The function of primary cilia in most tissues is unknown. In the kidney they are mechanosensitive organelles that detect fluid flow through the tubule lumen [16]. In the liver primary cilia are present on cholangiocytes and they function as mechano-, osmo-, and chemosensors in intrahepatic bile ducts. Mutations in genes encoding cholangiocytes' ciliary-associated proteins result in cholangiociliopathies [17].

In recent years, growing attention on cilia has stimulated the creation of numerous databases [18,19] including genomic and proteomic data on cilia composition [20-22].

## Functions involving cilia

### Motility

Many studies have demonstrated that motility is one of the main functions of cilia, and its impairment may cause severe phenotypes. In the absence of Ktu/PF13, both outer and inner dynein arms are missing or defective in the axoneme, leading to a loss of motility [23]. A murine mutation of the adenylate kinase 7 (*Ak7*) gene results in animals presenting with ultrastructural ciliary defects and decreased ciliary beat frequency in the respiratory epithelium. Ak7 seems to be a marker for motile 9+2 cilia and to have a crucial role in maintaining ciliary structure and function. The enzymatic activity may be involved in the energy production process necessary for movement of 9+2 cilia [24].

The mouse *Fu *(*Stk36*) gene is an effector of Hedgehog (Hh) signaling. Recently, Wilson and colleagues have demonstrated that mouse Fu is essential for construction of the central pair apparatus of motile 9+2 cilia. These studies have identified common regulators of Hh signaling and motile ciliogenesis [25].

Ciliary motility is also required for brain development and function. The ependymal motile 9+2 cilia are responsible for ependymal flow. Loss of ependymal cilia motility leads to an impaired fluid flow in the brain ventricles, resulting in hydrocephalus [26-28].

Motility is the main feature of the unique 9+0 primary cilium at the embryonic node, which is essential for correct embryonic development [3]. In the *Kif3a *null mutant mice, the node lacked monocilia while the basal bodies were present. These mice survive beyond mid-gestation, exhibiting growth retardation, pericardial sac ballooning, and neural tube disorganization. In addition, mutant embryos showed randomized left-right asymmetry and randomized turning and heart looping [3].

### Cell cycle

Centrioles play a dual role in the cell. They form the centrosomes that can interconvert with basal bodies upon ciliation. At the same time, they also give rise to the poles of the mitotic spindle.

Recent advances have demonstrated that ciliary proteins are involved in the regulation of the cell cycle. Mutations in IFT genes have clearly demonstrated a correlation between primary cilia and cell cycle control. Inactivation of IFT88 by RNAi in HeLa cells promoted cell cycle progression whereas IFT88 overexpression prevented G1-S transition [29]. The NEKs (never in mitosis gene A related kinases) are ciliary proteins, and mutations in *Nek1 *and *Nek8 *cause polycystic kidney disease (PKD) in murine models [30]. Activation of the centrosomal Aurora A kinase, which promotes mitotic entry in mammalian cells, induced the rapid reabsorption of cilia through tubulin deacetylation [31]. *PKD1 *and *PKD2 *are mutated in the autosomal dominant form of PKD [32,33]. They encode for Polycystin 1 (PC1) and Polycystin 2 (PC2), respectively, and are both involved in the regulation of the cell cycle. In response to fluid flow, the C-terminal tail of the plasma membrane PC1 is proteolytically removed and the protein translocates to the nucleus and directly initiates signaling processes linked to proliferation [34]. Overexpression of PC1 induces p21 expression and directly activates the JAK/STAT (Janus kinase/Signal Transducer and activator of transcription) signaling [35]. PKD2 interacts with ID2 (inhibitor of DNA binding 2) and regulates cell proliferation and differentiation [36].

Misregulation of cell cycle control is at the basis of oncogenesis. The cancer-promoting proteins Aurora A and HEF1/NEDD9/CAS-L have a role in primary cilium stabilization. Loss of cilia in cancer may contribute to the insensitivity of cancer cells to environmental repressive signals (reviewed in [37]). Although mutations in ciliary proteins do not predispose to cancer, it is fascinating to speculate that alterations in cilia, coupled to other mechanisms, might have a role in the development of cancer.

## Signal transduction

**Figure 2:**
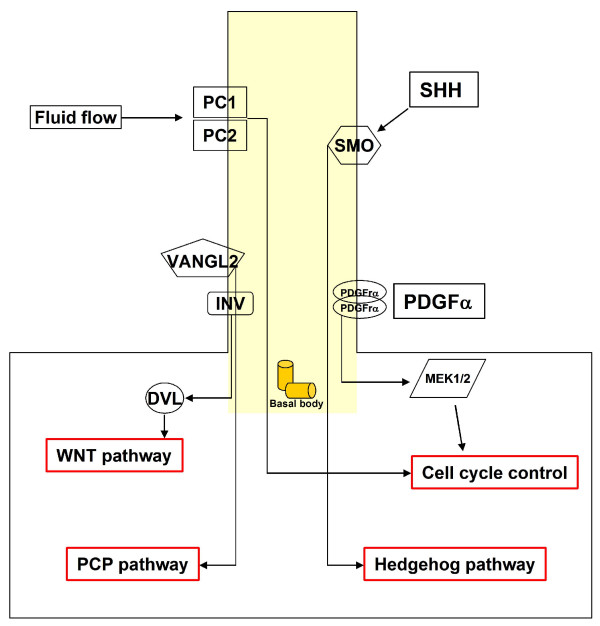
**A summary of the pathways transduced by the primary cilium**. The primary cilium is indicated in yellow with its basis formed by the basal body. In response to fluid flow, Polycystin 1 (PC1) and Polycystin 2 (PC2) are able to control the cell cycle. The platelet-derived growth factor alpha (PDGFα) signaling controls cell cycle and is transduced through the primary cilium. Vangl2 and Inv localize to primary cilia. Vangl2 is important for the planar cell polarity (PCP) pathway. Inv is the molecular switch between the PCP and Wnt pathways. Sonic hedgehog (Shh) signaling acts through Smoothened (Smo) that localizes to the primary cilium.

Cilia are unique organelles, which can act as antennae for the cells. They have crucial roles in several signal transduction pathways such as Hh, Wnt, planar cell polarity (PCP) and platelet-derived growth factor (PDGF) pathways. A summary of the pathways is reported in Figure 2.

### Hedgehog pathway

The evolutionary conserved Hh pathway regulates many developmental processes. The main players of the pathway are the three ligands, Sonic hedgehog (Shh), Indian hedgehog (Ihh) and Desert hedgehog (Dhh), the two membrane receptors, Patched1 (Ptc1) and Smoothened (Smo), and the effectors Gli transcription factors. In the absence of ligands, the transmembrane Ptc1 protein inhibits Smo to transduce the signal and Gli3 is constantly proteolytically cleaved into the repressor form Gli3R. The binding of the ligand to Ptc1 induces the release of Smo, which in turn inhibits Gli3 processing. As a result of Hh pathway activation, the Gli3 activator (Gli3A) regulates the downstream targets.

Several studies support the relationship between primary cilia and the Hh pathway [38-41]. In mammalian cells, Smo-dependent signaling requires translocation to primary cilia. Activation of the Shh pathway markedly upregulates the localization of Smo at the primary cilium in mouse embryonic fibroblasts and Madin-Darby Canine Kidney cells [38,39]. In the absence of Shh signaling, Ptc1 localizes to primary cilia and prevents Smo accumulation within cilia. When Shh signaling is activated Shh binds to Ptc1 and Ptc1 leaves the cilia, leading to accumulation of Smo. Thus, primary cilia function as chemosensors for the detection of extracellular Shh [40]. A crucial role in signaling was demonstrated for beta-arrestin 1 or beta-arrestin 2. Their depletion prevented localization of Smo to primary cilia and Smo-dependent activation of Gli [41].

Among many other functions, the Shh pathway is required to specify a set of ventral cell types in the developing neural tube. Mutations in two proteins of the IFT complex B, Ift172 and Ift88, caused the same specification defects in the neural tube observed in *Shh *mutants [42]. The neural phenotype of double mutant embryos (*Patched1/Ift172 *and *Patched/Ift88*) is identical to the single *Patched1 *mutant, indicating that IFT proteins act downstream of Ptc1. Inactivation of the *Ofd1 *gene, which encodes for a centrosome/basal body protein, showed neural tube defects similar to those observed in *Shh *mutants and Gli3 processing was shown to be altered in these mutant mice, suggesting that it may act through Shh signaling ([43] and BF, unpublished results). Moreover, IFT proteins are required for Gli activation and for the proteolytic processing of Gli3A in Gli3R [39,42,44-46]. Gli2 and Gli3 proteins, as well as Suppressor of Fused (SuFu), a negative regulator in the Hh signaling pathway, localize to the tip of cilia in primary limb-bud cell cultures [44]. Furthermore, recent studies have showed that IFT-dependent trafficking of Hh pathway components through the cilium is essential for their function [47].

### Wnt pathway

Cilia transduce signals for another important pathway. Wnt signaling is generally divided into two transduction pathways: the canonical Wnt and the non-canonical, known also as the PCP pathway. Wnt proteins bind to 'frizzled' receptors, leading to downstream activation of gene transcription by β-catenin, which is the major player of the pathway. Another key protein of the Wnt pathway is Dishevelled (Dvl), which localizes both to the cytoplasm and to the membrane. Degradation of the cytoplasmic Dvl by Inversin causes the switch to the PCP pathway. Thus, Inversin is considered the molecular switch between the two pathways [48].

The evolutionarily conserved PCP pathway drives several important cellular processes, including epithelial cell polarization, cell migration and mitotic spindle orientation. Molecular key components of the PCP pathway localize to the primary cilium. In addition to cell-cell junctions and the nucleus, Inversin localizes to the primary cilia of kidney epithelial cells, fibroblasts and pituitary gland [49,50]. Vangl2 localizes to the primary cilium in collecting duct cells and in human respiratory cells [51]. In the kidney Vangl2 genetically interacts with Bardet-Biedl syndrome (BBS) genes. Mutations in components of the PCP pathway lead to neural tube defects, open eyelid and disrupted cochlear stereociliary bundles [52,53]. Disruption of *Xenopus laevis *orthologs of the *Drosophila melanogaster *PCP effectors inturned (in) or fuzzy (fy) affected both PCP-dependent convergent and also elicited embryonic phenotypes consistent with defective Hh signaling [54].

Two studies have demonstrated that the primary cilium has a role in restraining Wnt/β-catenin signaling [55,56]. The basal body is an important regulator of Wnt signal interpretation and defects in this system may contribute to phenotypes of human ciliopathies [55]. In addition, the anterograde motor subunit Kif3a restrains canonical Wnt signaling [56].

Recently, Park and co-workers [57] have demonstrated that Dvl is essential for the apical positioning of basal bodies. Dvl and Inturned mediate the activation of Rho GTPase specifically at basal bodies, and the three proteins together mediate the docking of basal bodies to the apical plasma membrane. Once docked, basal bodies again require Dvl and Rho for the planar polarization that underlies directional beating of cilia, suggesting that a common signaling apparatus governs both apical docking and planar polarization of basal bodies [57].

Another molecular link between ciliary signals and Wnt pathways is the *Seahorse *gene required for establishing left-right asymmetry and for preventing kidney cyst formation in zebrafish. Seahorse is a cytoplasmic protein which constrains the canonical Wnt pathway and promotes the non-canonical Wnt pathway during gastrulation [58].

### PDGF pathway

The primary cilium in fibroblasts plays a critical role in growth control via the PDGF signaling pathway that is involved in numerous processes during development including proliferation, survival and migration. Activated PDGF receptors induce activation of MEK1/2-ERK1/2 (mitogen-activated protein kinase (MAPK)-extracellular signal regulated kinase (ERK)) pathways. The PDGF receptor α (PDGFRα) localizes to the primary cilium during growth arrest in NIH3T3 cells and primary cultures of mouse embryonic fibroblasts [59]. As a consequence of the interaction between the ligand (PDGF-AA) and the receptor (PDGFRα), the PDGFRα receptor becomes phosphorylated and phosphorylates Mek1/2, within the cilium.

## Human ciliopathies

Mutations in human ciliary genes give rise to a wide spectrum of disorders named ciliopathies. These include different types of disease from embryonic lethal to less severe multisystemic disorders (reviewed in [60-62]). Primary cilia are multi-subunit complexes composed of hundreds of proteins, and the inactivation of only one of these may be sufficient to produce a defective cilium or a complete immature cilium, resulting in variability of phenotypic severity. Many organs, and corresponding phenotypes, may be affected in ciliopathies, suggesting a tissue-specific function of primary cilia. Furthermore, many ciliary genes are mutated in more than one ciliopathy, indicating an interaction among proteins. Ciliary proteins may also have other roles in addition to those strictly connected to cilia. All these observations together shed light on the complexity and variety of ciliopathies. A list of ciliary genes whose mutations are responsible for human genetic disorders is reported in Table 1.

**Table 1:** Ciliary proteins associated with human genetic disease.

Human Disease	Hereditary transmission	Disease pathology	OMIM number	Gene symbol	References
Primary Ciliary Dyskinesia	Autosomal recessive	Respiratory infections, anosmia, male infertility, otitis media and situs inversus	604366	*DNAI1*	[67]
			603335	*DNAH5*	[68]
			603339	*DNAH11*	[69]
			612444	*DNAI2*	[70]
			607421	*TXNDC3*	[71]
			612650	*RSPH9*	[72]
			612650	*RSPH4A*	[72]

Meckel-Gruber syndrome	Autosomal recessive	Brain malformation, polydactyly, kidney and liver cysts	249000	*MKS1, BBS13*	[79]
			607361	*MKS3, TMEM, JBTS6*	[81]
			611134	*MKS4, CEP290, JBTS5*	[78]
			611561	*MKS5, RPGRIP1L, JBTS7*	[80]
			612013	*MKS6, CC2D2A*	[77]

Autosomal dominant form of polycystic kidney disease	Autosomal dominant	Polycystic kidney	601313	*PKD1*	[32]
			173910	*PKD2*	[33]

Autosomal recessive form of polycystic kidney disease	Autosomal recessive	Polycystic kidney	606702	*PKHD1*	[88]

Nephronophthisis type 1 **(**juvenile**)**	Autosomal recessive	Kidney cysts, liver fibrosis, retinal dysplasia	607100	*NPHP1*	[100]
Nephronophthisis type 2 **(**infantile**)**			602088	*NPHP2, INV*	[96]
Nephronophthisis type 3 **(**adolescent**)**			608002	*NPHP3*	[95]
Nephronophthisis type 4			607215	*NPHP4*	[101]
Nephronophthisis type 5			602937	*NPHP5/IQCB1I*	[97]
Nephronophthisis type 6			610142	*NPHP6/CEP290*	[99]
Nephronophthisis type 7			611498	*NPHP7, GLIS2*	[98]
Nephronophthisis type 8			610937	*NPHP8, RPGRIP1L*	[80]; [102]
Nephronophthisis type 9			609799	*NPHP9, NEK8*	[103]

Joubert syndrome 1	Autosomal recessive	CNS abnormalities, kidney cysts, brain and retina malformations	608629	*AHI1*	[108]
Joubert syndrome 4			609583	*NPHP1*	[107]
Joubert syndrome 5			610188	*NPHP6/CEP290*	[110]
Joubert syndrome 6			610688	*MKS3, TMEM, JBTS6*	[84]
Joubert syndrome 7			611560	*MSK5, RPGRIP1L, JBTS7*	[80]; [109]

Retinitis pigmentosa 1	Autosomal recessive	Retinal degeneration	180100	*RP1*	[117]
Retinitis pigmentosa 3	X-linked recessive		300389	RPGR*	[118]

Senior-Loken syndrome 1	Autosomal recessive	Renal dysfuntions and retinal degeneration	266900	*NPHP1*	[119]
Senior-Loken syndrome 4			606996	*NPHP4*	[101]
Senior-Loken syndrome 5			609254	*NPHP5/IQCB1*	[97]
Senior-Loken syndrome 6			610189	*NPHP6/CEP290*	[99]

Oral-facial-digital syndrome type I	X-linked dominant	Malformations of the face, oral cavity and digits, kidney cysts	311200	*OFD1*	[121]

Bardet-Biedl syndrome	Autosomal recessive	Kidney cysts, obesity, anosmia, retinal dystrophy, male infertility, situs inversus, diabetes	209901	*BBS1*	[128]
			606151	*BBS2*	[123]
			608845	*BBS3, ARL6*	[131]
			600374	*BBS4*	[130]
			603650	*BBS5*	[127]
			604896	*BBS6, MKKS*	[124]
			607590	*BBS7*	[129]
			608132	*BBS8, TTC8*	[126]
			607968	*BBS9, PTHB1*	[133]
			610148	*BBS10*	[125]
			602290	*BBS11, TRIM32*	[132]
			610683	*BBS12*	[134]
			609883	*BBS13, MKS1*	[135]
			609883	*BBS14, CEP290*	[135]

Almström syndrome	Autosomal recessive	Retinitis pigmentosa, deafness, obesity and diabetes mellitus	203800	*ALMS1*	[143,144]

*Note that Moore and colleagues found mutations in the RPGR gene in patients affected by both primary ciliary dyskinesia (PCD) and retinitis pigmentosa (RP) indicating an X-linked transmission of PCD [76].

Mutations in genes encoding for ciliary proteins, leading to immotile cilia or disrupted movement of cilia, are responsible of a set of human diseases named primary ciliary dyskinesia (PCD; OMIM: 242650). Patients with PCD may suffer from respiratory infections, anosmia, male infertility, otitis media and situs inversus and have been shown to have defects in ciliary structure and function ([63] and reviewed in [64]). Male infertility can be caused by loss of sperm flagellar motility, and some patients with PCD also display hydrocephalus. Respiratory infections are mainly caused by defective mucociliary clearance, which requires the presence of motile 9+2 cilia in respiratory epithelial cells.

PCD presents extensive locus heterogeneity [65] and only few genes, accounting for 40% of patients, have been identified to date for this disease. A successful approach for localization of putative PCD genes has been a homozygosity mapping strategy, which allows the localization of the *DNAH5 *gene [66]. Unfortunately, large informative families are rarely available. PCD affects motile cilia that are characterized by the presence of motor molecules within the ciliary axoneme. Inner (IDAs) and outer dynein arms (ODAs), together with radial spokes and nexin links, are necessary for cilia movement. Individuals with PCD generally present with mutations in one of the above motor molecules. Mutations in DNAI1, a dynein intermediate chain, and in DNAH5, encoding for one of the ODA heavy chains, have been associated with PCD [67,68]. Furthermore, mutations in the DNAH11 axonemal dynein heavy chain [69] and in the DNAI2 protein have been identified [70]. *TXNDC3*, encoding for a thioredoxin-nucleoside diphosphate kinase [71], *RSPH9 *and *RSPH4A*, encoding for radial spoke head proteins, were also found to be mutated in patients with PCD [72].

Furthermore, several dynein chain genes have been analyzed for mutational analysis in patients with PCD without success. Mutational analysis was negative for both the *DNAH9 *[73] and the *DNAL1 *[74] genes.* DNAH1 *was proposed as a candidate gene for human PCD [75] but to date no study has validated this hypothesis. PCD is classically transmitted as an autosomal recessive trait. Moore and colleagues have also demonstrated an X-linked transmission of PCD, where *RPGR *mutations were found in patients with a complex X-linked phenotype combining PCD and retinitis pigmentosa [76].

Meckel-Gruber syndrome (MKS; OMIM 249000) is an autosomal recessive lethal disorder characterized by renal and hepatic cysts, polydactyly, malformations in the central nervous system (CNS), and occasionally hydrocephalus. To date, mutations in five genes responsible for the disease have been identified [77-81]. Several MKS fetuses presented mutations in BBS genes, indicating possible genetic interaction between MKS and BBS genes [82]. In addition, most of the MKS genes are also mutated in other ciliopathies such as Joubert syndrome, suggesting a genetic interaction between ciliary genes [78,80,83,84].

Among ciliopathies that mainly affect the kidney the PKDs, which include autosomal dominant PKD (ADPKD), autosomal recessive PKD (ARPKD) and nephronophthisis (NPHP), are worth mentioning. Correlations between genotype and phenotype in ADPKD and ARPKD were reviewed by [85]. PKD1 and PKD2 proteins are multi-pass integral membrane proteins that interact to form a channel for the Ca^2+ ^ion [86]. Intracellular Ca^2+ ^levels, which are important for cell proliferation, apoptosis and ion reabsorption rates, may contribute to renal cyst formation [87]. *PKHD1*, the gene mutated in ARPKD (OMIM 263200), encodes for the membrane-associated receptor-like protein fibrocystin/polyductin [88]. PKHD1 associates with primary cilia of epithelial cells and co-localizes with PKD2. Recently, *in vivo *studies have demonstrated that the two proteins may function in a common molecular pathway [89]. To date, the molecular mechanisms considered responsible for kidney cysts are increased cell proliferation and/or loss of cell polarity [90-92].

NPHP (OMIM 256100) is an autosomal recessive cystic renal disease (reviewed in [93,94]). Individuals with this disease can also suffer from situs inversus, pancreatic and hepatic fibrosis, retinal degeneration (in Senior-Løken syndrome (SLSN) and Joubert syndrome (JBTS)), complex brainstem malformation and mental retardation (JBTS). In contrast with PKD, NPHP shows normal or diminished kidney size, cysts are concentrated at the corticomedullary junction, and tubulointerstitial fibrosis is dominant.

To date, mutations in nine genes linked to NPHP have been identified [95-103]. Nephrocystins, the proteins encoded by NPHP genes, are highly conserved in evolution. Mutations in NPHP genes cause defects in signaling mechanisms, including the non-canonical Wnt signaling pathway. *NPHP1 *encodes for nephrocystin-1, a protein that interacts with components of cell-cell and cell-matrix signaling, including p130Cas, focal adhesion kinase 2, tensin, and filamin A and B. Mutations in this gene causes juvenile NPHP type1 [100]. Mutations in the *Inversin *gene cause NPHP type 2 [96]. The *inv *murine model presents a complete inversion of left-right asymmetry and pancreatic and renal cysts [104]. Recently, Shiba and colleagues have demonstrated that the Inv protein is localized at a distinctive proximal segment of the primary cilium [105].

Mutations in *NPHP3 *are responsible for adolescent NPHP type 3 [95]. Mutations in the murine ortholog *Nphp3 *cause the renal cystic mouse mutant *pcy*. *NPHP4 *is mutated in NPHP type 4 [101]. The encoded protein, nephrocystin-4/nephroretinin, forms a complex with other proteins involved in cell adhesion and actin cytoskeleton organization, such as nephrocystin-1, p130Cas, Pyk2, tensin, filamin, and α-tubulin. *NPHP5*, mutated in NPHP type 5, encodes the protein nephrocystin-5 [97]. All patients had early onset retinitis pigmentosa (SLSN). Nephrocystin-5 contains two IQ domains, which directly interact with calmodulin and form a complex with the retinitis pigmentosa GTPase regulator, which, when defective, causes X-linked retinitis pigmentosa. Both nephrocystin-5 and retinitis pigmentosa (RP) GTPase regulator (RPGR) localize to connecting cilia of photoreceptors and in primary cilia of renal epithelial cells. The fact that connecting cilia of photoreceptors are the structural equivalents of primary cilia of renal epithelial cells may explain retinal involvement in the retinal-renal syndrome SLSN.

*NPHP6/CEP290 *encodes a centrosomal protein and is the cause of NPHP type 6 and JBTS type 5. Abrogation of *NPHP6 *function in zebrafish causes PCP (convergent extension) defects and recapitulates the human phenotype of NPHP type 6, including renal cysts, RP, and cerebellar defects. Nephrocystin-6 is expressed in the centrosomes and mitotic spindle in a cell-cycle-dependent manner. Its identification establishes a link between centrosome function and tissue architecture in the pathogenesis of cystic kidney disease, RP, and CNS development. Mutations in *NPHP6/CEP290 *have been confirmed to cause JBTS with or without renal involvement [99]. It is interesting that a 300-amino acid in-frame deletion of *NPHP6/CEP290 *caused retinal degeneration alone, without renal or cerebellar involvement, in the *rds16 *mouse model. This is in accordance with the recent finding that a hypomorphic mutation of *NPHP6/CEP290 *represents the most frequent cause of Leber's congenital amaurosis. The seventh gene identified in NPHP is *GLIS2*, which encodes for a transcription factor [98]. The murine model of Glis2 presented severe renal atrophy and fibrosis of the kidney. The essential role of Glis2 is the maintenance of renal tissue architecture through prevention of apoptosis and fibrosis [98]. Recently, mutations in the gene *RPGRIP1L *[102] and *NEK8 *were found in patients affected by NPHP [103].

JBTS is a ciliopathy characterized by extensive genetic heterogeneity and variability in phenotypic severity (reviewed in [106]). To date, five genes linked to JBTS have been identified [80,84,107-111]. Children with JBTS appear to have a characteristic facial appearance, delayed language, autism, polydactyly, renal cysts, microcephaly and ocular abnormalities. JBST1 and JBST2 loci have been mapped but no causative genes have yet been identified. The gene responsible for JBTS type 3 is *AHI1*, which encodes for jouberin that contains an N-terminal coiled domain [108]. The gene for JBTS type 4 was identified in patients presenting with juvenile NPHP [107]. The gene mutated in patients with JBTS type 5 encodes for the centrosomal protein CEP290 [110].

Cilia also have a major role in the retina. Defects in their function can cause retinal ciliopathies (reviewed in [112]). Photoreceptors are composed of an outer and an inner segment connected by a highly specialized 9+0 cilium called 'connecting cilium'. The two proteins RP1 and RPGR associated with RP both localize predominantly to the photoreceptor connecting cilium [113,114]. The microtubule-associated protein RP1 is mutated in RP type 1 (OMIM 180100) [115-117]. Mutations in RPGR are associated with RP type 3 (OMIM 180100) [118]. Moreover, in ciliopathies retinal degeneration is often one of the major phenotypic features. In particular, in SLSN (OMIM 266900), a rare autosomal recessive disorder, patients are affected by NPHP and progressive eye disease. The genes responsible for SLSN are *NPHP1*, *NPHP4*, *NPHP5/IQCB1 *and *CEP290*, which are the same genes mutated in other ciliopathies such as NPHP and JBTS [97,99,101,119].

Oral-facial-digital type I syndrome (OFDI; OMIM 311200) is an X-linked dominant developmental disorder with lethality in males. Female patients present malformations of the oral cavity, face, digits and CNS malformations. *OFD1*, the gene responsible for this genetic disorder, encodes a protein localized at the centrosome/basal body [120,121]. *Ofd1*-knockout animals reproduce the main features of the human disease, albeit with increased severity, possibly due to differences of X-inactivation patterns between human and mouse [122]. Inactivation of the gene indicated that OFD1 is required for primary cilia formation and left-right axis specification [43]. About 50% of patients with OFDI present CNS abnormalities (agenesis of corpus callosum, intracerebral cysts/porencephaly, gray matter heterotopias, and cerebellar malformations). Interestingly, about 50% of patients present mental retardation, which in some cases is not accompanied by gross structural abnormality, suggesting a role for primary cilia in the maintenance of adult organs.

BBS (OMIM 209900) is a genetically heterogeneous pleiotropic disorder with symptoms including kidney and gonadal abnormalities, mental retardation, retinal degeneration, obesity, diabetes, and polydactyly. To date, fourteen BBS genes have been identified whose mutations are associated with the syndrome [123-135]. BBS exhibits a complex pattern of inheritance, the triallelic inheritance, in which three mutations at two loci simultaneously are necessary and sufficient in some families to manifest the phenotype [136]. The genetic and cellular characteristics of BBS have recently been extensively discussed [137]. Most of the BBS proteins localize to the cilia/basal body/centrosome complex. BBS proteins interact in a multi-subunit complex that is proposed to regulate RAB8-dependent vesicular trafficking of membrane proteins from the Golgi to the ciliary membrane [138]. Moreover, studies conducted both in humans and in several other model systems have indicated that BBS proteins act in microtubule-based cellular processes [139-142]. Studies in *Caenorhabditis elegans *revealed that BBS-7 and BBS-8 are required to keep IFT particles intact [139]. Morpholino knock-down of BBS genes in zebrafish resulted in a delay of retrograde intracellular transport of melanosomes with melanophores [140]. RNAi silencing of BBS4 causes PCM1 mis-localization, de-anchoring of microtubules at the centrosome and arrested cell division [141]. Patients with BBS can suffer from anosmia and, interestingly, olfactory cilia of *Bbs1 *and *Bbs4 *mutant mice are depleted of stable microtubules [142].

Almström syndrome (ALMS; OMIM 203800) is a disorder similar to BBS since patients are affected by obesity, diabetes and retinal degeneration. In addition they show sensorineural deafness, cardiomyopathy, liver dysfunction and kidney dysfunction, but do not have polydactyly. To date, one gene has been identified as responsible for this disease [143,144]. *ALMS1 *encodes for a protein that localizes both to the basal body and centrosomes [145]. *In vitro *studies have demonstrated that ALMS1 is important for ciliogenesis and that it has a role in mechanosensation, since inactivation of the gene resulted in the prevention of Ca^2+ ^influx into the cytosol [146].

## Cilia in development and adult life: do they play the same role?

All the pathways described, together with primary cilia function, have crucial roles in developmental processes (reviewed in [62,147]). Cilia may have diverse cellular functions during both development and adult tissue homeostasis. Among cilia developmental defects, intraflagellar transport proteins play crucial roles and they contribute to the establishment of left-right asymmetry. The transgene insertion of the *Ift88 *(*Tg737 *or *polaris*) allele in homozygosity caused PKD and preaxial polydactyly [26]. The hypomorphic mutants *Ift88*^*orpk *^died within two weeks of birth, showed growth defects and were affected by hydrocephalus [26]. Complete inactivation of the *Ift88 *allele caused lethality at midgestation [148]. The mutant embryo showed randomized left-right asymmetry associated with loss of cilia in the node.

Furthermore, inactivation of the *Tbx6 *gene led to randomized turning and heart looping. The mutation had a severe effect on the morphology and motility of nodal cilia, demonstrating that Tbx6 is essential for correct left/right axis determination in the mouse and acts through effects on Notch signaling around the node as well as through an effect on the morphology and motility of nodal cilia [149].

The Ift122 protein is a component of IFT particle A. *Ift122 *null embryos show multiple developmental defects that result in lethality. In the node, primary cilia were absent or malformed in homozygous mutant and heterozygous embryos, respectively [150].

A mouse mutation, hennin (hnn), caused coupled defects in cilia structure and Shh signaling [151]. The hnn murine mutant model showed defects in the neural tube with a Shh-independent expansion of the domain of motor neuron progenitors. The hnn mutation is a null allele of Arl13b, a small GTPase of the Arf/Arl family, and the Arl13b protein is localized to cilia [151].

Primary cilia are required for cerebellar, hippocampus and forebrain development. Specific inactivation in the CNS of *Tg737 *and *Kif3a *causes severe cerebellar hypoplasia and foliation abnormalities, primarily attributable to a failure of expansion of the neonatal granule cell progenitor population [152]. In addition, recent studies have demonstrated that *Kif3a *is essential for Shh-dependent expansion of cerebellar progenitors [153]. Conditional ablation of the gene in cells derived from Cre-expressing cells under the human glial fibrillary acidic protein promoter resulted in loss of primary cilia in cerebellar granule cell precursors (GCPs). In this animal model, GCPs were specified, but a severe defect in late embryonic and early postnatal expansion of GCPs resulted in atrophied cerebella [153]. The same animal model was analyzed for production of adult neural stem cells in the hippocampus and also revealed the absence of primary cilia in the developing dentate gyrus. Primary cilia and Shh signaling are essential for the expansion and establishment of granule neuron precursors in the post-natal dentate gyrus [154].

Mutant mice for *Stumpy *lack cilia and have evident abnormalities in post-natal developing brain regions, including a hypoplasic hippocampus characterized by a primary deficiency in astrocyte-like neural precursors [155].

Cobblestone is a hypomorphic allele of the IFT gene *Ift88*. Cobblestone mutants show both severe defects in the formation of dorsomedial telencephalic structures, such as the choroid plexus, cortical hem and hippocampus, and also a relaxation of both dorsal-ventral and rostral-caudal compartmental boundaries. In this animal model, Gli3 proteolytic processing is reduced and an upregulation of canonical Wnt signaling in the neocortex and in the caudal forebrain has been observed. These results indicate a critical role for ciliary function in the developing forebrain [156]. In addition, the inactivation of *Ift172 *revealed that it is required in the patterning of the mammalian brain, and it plays a crucial role in primary cilia formation during development [157].

On the basis of all these studies, the role of cilia in development is important in defining the structure of the organism. In fact, impairment in cilia function leads to structural defects (polydactyly, brain abnormalities, left-right asymmetry). Several ciliopathies such as Bardet-Biedl, Almström syndrome, Joubert and oral-facial-digital syndrome type I are pleiotropic disorders, which include limb abnormalities, renal cystic disease, CNS abnormalities including mental retardation, and/or obesity. In several cases, however, mental retardation is not associated with CNS structural abnormalities. Obesity and mental retardation, not associated with structural defects, can be considered behavioral defects. This observation suggests that cilia may have an important role in organ maintenance and function, yet to be defined, besides the well-established role during development.

For this reason, an intriguing issue is the role of cilia in adult life, when all the structures are already defined. To date, little is known about the role and importance of cilia during post-natal life. It would be fascinating to speculate about developmental defects in congenital ciliopathies and defects of adult-onset ciliopathies. The role of the ciliary gene *Pkd1 *during post-natal life has been investigated [158]. Inactivation of *Pkd1 *in mice before day 13 resulted in severe polycystic kidney, while inactivation at day 14 and later resulted in slow-onset cystic kidney disease. These studies revealed a temporal breakpoint for cyst formation in the kidney and may reflect the different function of Pkd1 and cilia in the adult organ with respect to the developing one [158].

Moreover, recent studies have shown that inactivation of two ciliary genes *Tg737 *and *Kif3a *in adult life leads to obesity and slow-onset cystic kidney disease [159]. In order to better investigate the role of cilia in obesity, Davenport and colleagues [159] produced two additional mouse models, disrupting *Kif3a *in the neurons of the CNS and in the hypothalamus. Interestingly, loss of cilia on neurons resulted in hyperphagia and obesity, indicating that neuronal cilia have a main role in satiety responses [159].

Deletion of *Tg737 *from cells in the ovary by *Prx1*-Cre activity, which is detected in the follicle cells of the ovary, demonstrated that primary cilia have an essential role in ovarian function. Mutant mice showed abnormalities in the estrous cycle, alterations in ovulation, and a delay in mammary gland development, characterized by a lack of terminal end buds [160].

Recently, expression studies of components of the Hh pathway in the developing pancreas and in adult cancer pancreatic cell lines have demonstrated that the onset of Hh signaling from human embryogenesis to fetal development is associated with accumulation of Smo and Gli2 in duct primary cilia and with reduction of Gli3 in the duct epithelium [161]. Smo, Ptc1, and Gli2 localized to primary cilia of two pancreatic cancer cell lines, which may maintain high levels of non-stimulated Hh pathway activity. These findings indicate that primary cilia are involved in pancreatic development and post-natal tissue homeostasis [161].

Obesity in adult life is a main feature of the BBS human phenotype and BBS mouse models. Inactivation of *Bbs2*, *Bbs4 *and *Bbs6 *genes leads to obesity associated with increased food intake [162-164] and with hyperleptinemia and leptin resistance [165]. Recently, studies have demonstrated that BBS proteins are required for LepR signaling [166] and that a peripheral primary dysfunction of adipogenesis contributes to the pathogenesis of obesity in BBS [167].

The world of cilia is very complex and the studies carried out up to this point have demonstrated that cilia may have different roles in space and time. Further research is needed to dissect the role of cilia during adult life and to fill the gap in knowledge concerning adult-onset phenotypes.

## Conclusion

Remarkable advances in the understanding of cilia function have been made in a relatively short amount of time. Recent studies have provided key insights into the mechanisms involving cilia. These organelles appear to be involved in many cellular processes, such as proliferation, signal transduction and differentiation. The studies on primary cilia are revealing a complex scenario and their role may be organ-specific and even cell type-specific. This could explain the heterogeneity and complexity of phenotypes associated with mutations in ciliary genes.

To date, much attention has been focused on the role of primary cilia during development; recent studies are now shedding light on their function in post-natal life. Obesity and mental retardation seem to be prominent features of several ciliopathies. Understanding primary cilia function in adult life could help to elucidate the mechanisms responsible for such phenotypes, and further studies are needed in this direction.

Although the growing body of literature on primary cilia is promising, further studies are necessary to dissect and understand the primary cilium, a complex and dynamic organelle.

## Competing interests

The authors declare that they have no competing interests.

## Authors' contributions

AD and BF wrote the review manuscript. The authors read and approved the final manuscript.
